# Attitudes toward bone health among rural‐dwelling veterans identified as at risk of fracture: a qualitative analysis

**DOI:** 10.1002/jbm4.10501

**Published:** 2021-05-14

**Authors:** Jennifer M. Van Tiem, Melissa J.A. Steffen, Aaron T. Seaman, Karla Miller, Shylo E. Wardyn, Christopher C. Richards, Samantha L. Solimeo

**Affiliations:** ^1^ Veterans Affairs (VA) Office of Rural Health, Veterans Rural Health Resource Center‐Iowa City, Iowa City VA Health Care System Iowa City Iowa USA; ^2^ VA Health Services Research & Development Service, Center for Access and Delivery Research and Evaluation Iowa City VA Health Care System Iowa City Iowa USA; ^3^ Department of Internal Medicine Carver College of Medicine, University of Iowa Iowa City Iowa USA; ^4^ Department of Medicine Salt Lake City VA Health Care System Salt Lake City Utah USA; ^5^ Division of Rheumatology University of Utah School of Medicine Salt Lake City Utah USA

**Keywords:** AGING, HEALTH SERVICES RESEARCH, OSTEOPOROSIS, PATIENT‐CENTERED, PRACTICE‐RELATED ISSUES

## Abstract

Although much is known about system‐level barriers to prevention and treatment of bone health problems, little is known about patient‐level barriers. The objective of this study was to identify factors limiting engagement in bone health care from the perspective of rural‐dwelling patients with known untreated risk. Over 6 months, 39 patients completed a qualitative interview. Interview questions focused on the patient's experience of care, their decision to not accept care, as well as their knowledge of osteoporosis and the impact it has had on their lives. Participants were well‐informed and could adequately describe osteoporosis and its deleterious effects, and their decision making around accepting or declining a dual‐energy x‐ray absorptiometry (DXA) scan and treatment was both cautious and intentional. Decisions about how to engage in treatment were tempered by expectations for quality of life. Our findings suggest that people hold beliefs about bone health treatment that we can build on. Work to improve care of this population needs to recognize that bone health providers are not adding a behavior of medication taking to patients, they are changing a behavior or belief. Published 2021. This article is a U.S. Government work and is in the public domain in the USA. *JBMR Plus* published by Wiley Periodicals LLC on behalf of American Society for Bone and Mineral Research.

## INTRODUCTION

Osteoporosis is underdiagnosed, with few adults who are at‐risk of osteoporosis and fragility fracture being screened,^(^ ^1^, ^2^ ^)^ and undertreated, with patients who need bone health medication either not being prescribed bone health medication or not taking it.^(^ ^3^, ^4^ ^)^ Identification and treatment of osteoporosis is significantly lower among rural‐residing US military Veterans than it is among urban‐residing Veterans,^(^ ^5^, ^6^, ^7^ ^)^ which is likely attributable to rural Veterans' limited access to diagnostic imaging and specialty care consultations generally available only in larger urban medical centers. In 2016 the Veterans Affairs (VA) Office of Rural Health committed funds to support an innovative approach to delivering primary osteoporosis prevention to rural Veterans, the Rural Bone Health Team (RBHT). The RBHT co‐manages osteoporosis care with Veterans' primary care providers (PCPs) by assuming clinical ownership of bone health screening and management. This co‐management is achieved by leveraging existing telehealth technology (i.e., RBHT initiated telephone consults with rural primary care patients at high risk of osteoporosis) and electronic health record capabilities (i.e., e‐consults), facilitated by a care coordination agreement.

The RBHT delivery model is described in detail elsewhere,^(^ ^8^ ^)^ but in brief, the clinic uses the VA electronic medical record to identify patients at risk of osteoporosis and then provides direct care using telehealth. The RBHT notifies patients of their risk by US Postal Service (US mail) and assists patients in obtaining a dual‐energy x‐ray absorptiometry (DXA) screening from the nearest VA or community facility. The RBHT also coordinates the screening with upcoming VA appointments. DXA results are examined in concert with a patient's clinical and behavior risk factors and the osteoporosis diagnosis communicated with the patient by phone. Patients who wish to initiate therapy receive medications at low to no cost through the US mail service. The RBHT was designed to overcome systemic barriers to prevention and treatment of bone health problems in Veterans, including PCPs overburdened with other health concerns and unsure how to identify at‐risk Veterans, treat osteoporosis, reduce high costs, avoid long travel times, and circumvent often cumbersome connections with non‐VA community providers. Little is known about patient‐level barriers to prevention and treatment in US Veterans.

Although implementation of the RBHT was determined to be feasible, less than half of eligible Veterans accept care from RBHT.^(^ ^9^, ^10^ ^)^ Given that this clinic design reduces cost and travel barriers, the objective of this study was to identify other factors limiting engagement in bone health care from the perspective of patients with known untreated risk. In this article we report our analysis of qualitative interviews with Veterans who declined care from the clinic, those who accepted a DXA but declined to initiate medications, and those who completed a DXA and started pharmacotherapy. We found that breaking down barriers to accessing diagnostic care does not always lead to more utilization of the affiliated treatments, and that understanding when and how patients fall out of care pathways is key to improving patient engagement and increasing utilization of preventative treatments for fragility fracture related to osteoporosis.

## MATERIALS AND METHODS

We identified barriers to patient acceptance of care from the RBHT by evaluating qualitative interviews conducted with three groups of patients: (i) patients who declined a DXA, (ii) patients who had a DXA, but declined medication, and (iii) patients who had a DXA and accepted medication. This study received Institutional Review Board (IRB) approval from the University of Iowa IRB (IRB #201805721).

### Study design

Knowledge of how Veterans', especially men's, attitudes toward bone health care affects their decision making is scant. Although there are studies examining knowledge and other factors in the context of secondary prevention models,^(^ ^11^ ^)^ to our knowledge there are little to no data examining the patient experience in the context of a primary prevention model such as the RBHT. Thus, we used an “exploratory descriptive design,”^(^ ^12^ ^)^ including conducting a literature review,^(^ ^13^ ^)^ purposive sampling,^(^ ^14^ ^)^ semistructured interviewing, and thematic analysis.^(^ ^15^ ^)^

Interview questions focused on the patient's experience of care or their decision to not accept care from the RBHT as well as their knowledge of osteoporosis and the impact it may have had on their lives.

### Study sample and recruitment

Participants in this study were all patients who had been contacted by the RBHT. Eligibility criteria for receiving outreach from the RBHT included: rural residence, evidence of regular Veterans Health Administration (VHA) primary care, renal sufficiency for treatment, and age‐related risk.^(^ ^10^ ^)^ We used electronic health record data to ascertain patient completion of DXA and initiation of osteoporosis medications when indicated, in order to recruit patients in each of our subgroups. Target sample size was set at 75 overall, 25 per group, consistent with typical qualitative approaches to achieving data saturation (20–30 participants in a heterogeneous sample, and five participants per subgroup).^(^ ^14^ ^)^ Participants were sent a recruitment letter and then called by a study team member (Shylo E. Wardyn) to ensure receipt of the materials, review the forms, review the elements of consent, answer questions about the study, and schedule the interview. Participants who wished to participate but were unable to complete a phone interview due to disability (e.g., poor hearing) were sent a written questionnaire to complete and return.

### Data collection

Phone interviews were conducted over 6 months and audio recorded with consent from the participant. The interview guide was developed to elicit patient experiences with the RBHT at different points in the care process, and to understand their attitudes toward screening and treatment of bone health, their beliefs and knowledge about osteoporosis, and the relative importance of osteoporosis in the context of their overall health and care. The interview guide is available in Appendix S1. The written questionnaire, sent to participants who were unable to complete a phone interview, was based on the interview guide. Interviews lasted from 5 to 40 min (mean, 19 min) and were transcribed by trained transcriptionists and then uploaded into a qualitative data analysis software, MAXQDA (Verbi Software, Berlin, Germany).^(^ ^16^ ^)^

### Data analysis

Interview data were analyzed using theoretical thematic analysis^(^ ^15^ ^)^ to identify semantic patterns within the data related to the interview questions. The codebook was developed using a consensus process. First, deductive codes were derived from the interview guide. Three authors (Samantha L. Solimeo, Melissa J.A. Steffen, and Jennifer M. Van Tiem) each coded the same nine interviews independently to facilitate discussions about codifying coding definitions and coding choices. Following these discussions, the same three authors each separately coded 10 interviews using the codebook. Coded data were then reviewed for intersection frequency by code and interviewee group (e.g., accepted care and medication) to identify patterns for narrative synthesis.

## RESULTS

Thirty‐nine patients completed the interview. Of the 39 patients who participated in interviews, seven patients declined a DXA, 15 patients had a DXA but declined medication, and 17 patients had a DXA and accepted medication. There were three participants who declined a DXA from the RBHT and later reported interest in DXA during the qualitative interview. See Table 1 for an overview. Our sampling strategy was designed to identify differences between those who did and did not accept DXA and those who did and did not initiate treatment. Overall, participants were well‐informed and could adequately describe osteoporosis and its deleterious effects. Though some participants reported surprise at receiving a letter from the RBHT informing them of their risk of developing osteoporosis, their decision‐making around accepting or declining a DXA scan and treatment was both cautious and intentional. Often people who chose not to take a prescription were willing to take supplements like calcium and vitamin D, engage in exercise, and modify their diet to eat more calcium‐rich foods. Decisions about how to engage in treatment were tempered by expectations for quality of life. Our findings suggest that encouraging patients to engage in care requires becoming interested in their understanding of their health and accommodating what about their lives they hope to preserve as they age.

**TABLE 1:** Numbers of interview participants by care decisions

Participant characteristics	Overall (*n* = 39)	Men (*n* = 33)	Women (*n* = 6)
Declined DXA	7	6	1
Completed DXA, declined pharmacotherapy	15	12	3
Completed DXA, initiated pharmacotherapy	17	15	2

Abbreviation: DXA, dual‐energy x‐ray absorptiometry.

### DXA decision‐making

Participants who declined a DXA mentioned competing priorities related to comorbidities such as cancer, essential tremors, or chronic obstructive pulmonary disease (COPD). In addition, beliefs about perceived importance and susceptibility influenced some patients' decision making. One participant reported that he perceived osteoporosis was an important health problem, but only “for younger people below [the] age of 80+ years;” he said that his sense of the importance of osteoporosis changed “recently after being diagnosed [with] adrenal cancer, and being told that [he had] broken his back” (P6). He listed “pain relief and control” and “quality of life” as more important than osteoporosis and declined care from the RBHT “because of [his age];” he said, “I felt others would need help more than I” (P6). Another participant reported that he “felt like [the outreach letter from RBHT] didn't apply to [him].” He described his decision to forgo a DXA scan thusly:

[I] think my bones are pretty healthy…I'm physically active a lot. I've never broken a bone. Well, I have smashed up some fingers and stuff over the years. But that they—, I‐I mean, they certainly seem strong enough for me. You know, I still do some manual labor—not as much as used to—and I'm fit. I'm not overweight, and I think my bones are in pretty good shape. I drink milk with cereal a couple three times a week, have a varied diet, and I haven't had any problems with my bones. (P8)

Among those who completed the DXA, we identified three patterns of decision making: (i) those who accepted a DXA because the RBHT recommended it, (ii) those who accepted a DXA because they wanted more information about their bone health, and (iii) those who accepted a DXA for both reasons. Participants who accepted a DXA on the recommendation from a care provider, and because they wanted more information about their bone health, described a constellation of overlapping concerns. One participant, who was wheelchair‐dependent, reported how:

My wife…convinced me I probably should be worried more about my bone density even though I have no side effects at this time…it'd be terrible if I broke a hip. I hop out of my wheelchair and I go on my butt on the ground quite a bit to do outside things. And so, if I ever broke…my hips I'd really be in a mess so. I guess it's got me thinking I should pursue, at least, a little more information about where I'm headed. … You know, falls and everything are getting more possible with age. (P1)

As this comment illustrates, DXA participants factored in what they knew about how they had lived their lives and they anticipated their future health based on what they knew and the facts of their health as they saw it.

### Treatment decisions

Of the 32 interviewees whose DXA results indicated significant fracture risk, two declined to initiate an osteoporosis medication due to their or their community providers' beliefs about medications. Patients who declined osteoporosis medication justified this decision as an effort to preserve their existing health by protecting themselves against perceived medication side effects. One participant reported being aware of how [alendronate] “makes your bones grow and it makes ‘em brittle” (P3); another participant remembered how a friend took [the medication] and “got the Guillain‐Barré business on her whole right side and [was] messed up to this very day” (P4). Participants made the decision to decline medication based on information, and not always hearsay. As one person described:

I got tested. My bone density was truly low and…they sent me a prescription to fill…I took it to my drugstore and got a feed‐out on what the side effects might be with that particular drug that was recommended. And because it was a very severe number of side effects, at my age, I'm in my 80s right now … but that was a few years ago. I was still old then. But in any event, I chose not to do that. (P7)

The participant with a double leg amputation accepted a DXA scan but sought a second opinion about his treatment options from a community provider. He decided not to take medication and reported how:

[The RBHT] was saying that I had low density, soft bone tissue and I should start more calcium pills and this bone—I can't remember which— alendronate—or something…they subscribed that, and I read up on all the disadvantages and advantages of it. And I decided to get that second opinion from a private doctor bone scan. And so, I never did start that medication…I've been strong, and I've used my upper body for my legs. I've had arthritis in my shoulders and I just wanted to make sure, you know, taking that medication was the proper thing to do because of all the side effects I read about. (P1)

Other participants declined medication because they were concerned with the volume of medications they were already taking, and how those medications also came with side effects that made them sick. A person who had had two heart attacks recalled how:

I never have liked to take medicine. I have to take medicine currently for my heart. I had two heart attacks and so I take that with a lotta reluctancy, but I have to do it… at one point in time I was taking…a HANDFUL of medicines and come to find out, they were making me sick, and so then they had to re‐evaluate and say, ‘Okay, well, you don't have to take this one. You don't have to take this one,’ and so finally we got down to where I'm taking, I think, two pills in regards to my heart… so to increase doing more of it [for bone health], I would really have to be assured or really make sure that I had to do it. (P2)

Participants' concerns about side effects were less about isolated side effects of osteoporosis treatments. Rather, they were concerns about side effects *and* age, or *and* current physical limitations, or *and* side effects from other medications. For these participants, even if they perceived that the treatment would prevent bone health problems, the potential side effects only highlighted their existing health concerns and physical limitations.

The 17 participants who initiated a medication after consultation with the RBHT were also already doing some form of bone health treatment such as engaging in exercise, taking supplements (e.g., calcium, vitamin D), or eating to promote bone health (e.g., incorporating more calcium‐rich foods into their diet). During an interview with a participant who had been prescribed alendronate after a DXA and was currently taking the medication, we asked about their conversations with the RBHT. The complexity of their response reflects the balance that all participants reported trying to achieve when taking care of their bone health, in concert with multiple care providers (i.e., their PCP and the RBHT), and with an eye to maintaining their lifestyle. Regarding supplements, the participant reported that RBHT:

They asked me about calcium and what I was doing. I told ‘em I didn't drink milk and I eat a lotta cheese. I like cheese, and that seemed to be okay for, you know, as far as I've been getting some input on calcium and stuff, and I take One‐a‐Day…vitamins and I–, then, they also asked that I have plenty of vitamin D and I take 4000 units of vitamin D every day, and I was doing that before this, before this. That was recommended to me by my doctor. (P5)

The RBHT also spoke with the individual about exercise. He already had an active lifestyle as a rancher, working with horses. He reported that the team spoke with him about exercise to maintain bone health:

Yes, they talked some about [exercise], before they knew much about what I did…but I told them I was not interested in any special exercises or anything and, because I'm very active anyway. And they said, “Well, from what we hear, we don't think you need to. You have to just be careful. Make sure that you know that you're susceptible to–, at your age, and also because of your density–, of fractures.” (P5)

Reflecting on his engagement with treatment for bone health and looking forward, thinking about his prognosis, the participant reported:

Well, I believe they told me I would be getting another scan sometime this summer and I'm looking forward to it to see if we're maintaining my density and if it's helping or if it's not, then what direction we gotta go so I don't get all humped up or crooked or whatever, see what they can do. And my doctor is aware of what [the RBHT is] doing and I think he's interested in seeing what's happening when I get results and sharing them with him, which I intend to do and so on. I'm quite positive about it…[bone health is] very important to me as far as it's my structure and as long as I can keep my bones healthy and my muscles toned, I can be active and doing what I'm doing. (P5)

Decisions to accept or decline treatment, specifically medication, related to participants wanting to sustain their quality of life and safeguard the shape of their lives, habits, and jobs. Present in each of their reports was an awareness of their age and the mounting limitations of their aging bodies. Decisions around treatment and preparing to accommodate the side effects presented by the medications were necessarily balanced with accommodations participants were already making as they grew older.

## DISCUSSION

The RBHT was designed to reduce osteoporosis screening and treatment for rural‐dwelling Veterans. Despite making care more accessible, some Veterans with fracture risk declined DXA, and others completed DXA but declined to initiate medications known to reduce their fracture risk. We interviewed Veterans to understand their decision making to enhance the clinic design and to contribute to the growing literature on low utilization of bone health care services. In doing so we: (i) confirm the importance of health beliefs and provider referral to care in the patient's decisions, and (ii) illustrate opportunities to build upon the patient's existing health beliefs in future efforts to improve DXA and treatment rates.

For all participants, whether they accepted or declined care, their decision was informed by a desire to maintain their lifestyle. Participants who declined care framed their bone health as an adjunct to their overall health. A DXA or medication could be declined or avoided without compromising their current health status; sometimes participants declined medication because they perceived that medication might ultimately compromise their current health status. Most participants reported accepting care. Accepting care meant finding a treatment that balanced positive steps they were already taking with additional treatment indicated through conversation with the RBHT.

A recent qualitative metasynthesis^(^ ^13^ ^)^ identified 10 studies examining patient barriers to DXA. Although most subjects were White women and thus not necessarily representative of older Veterans, the findings there suggest that patient engagement with osteoporosis care is affected by: osteoporosis's asymptomatic nature; perceived age and gender stigma associated with an osteoporosis diagnosis^(^ ^17^, ^18^, ^19^, ^20^, ^21^, ^22^ ^)^; confusion about the DXA's purpose; the belief that osteoporosis is not a serious health concern; the relatively lower ranking of osteoporosis in relation to other health conditions; and the belief that osteoporosis fractures are not indicative of disease but arise from poor luck.^(^ ^13^, ^23^ ^)^

Low rates of osteoporosis medication initiation, adherence, and persistence are well documented, but there has been comparatively little attention paid to patient rationale underlying these patterns, particularly in the context of primary prevention approaches.^(^ ^24^ ^)^ Recent pharmacological research suggests that fear of rare side effects (i.e., atypical fracture or osteonecrosis of the jaw) is driving low initiation rates.^(^ ^25^, ^26^ ^)^ Recent qualitative research has identified how adherence and persistence with medication can be impacted by the confluence of patients' medication beliefs, including perceived ineffectiveness and fear of side effects, with patients' preference for “natural” treatments and lack of knowledge about the connection between osteoporosis and risk of fracture.^(^ ^22^, ^27^, ^28^ ^)^

Our findings confirm findings from these other qualitative studies and suggest that people hold beliefs about bone health treatment that we can build on. Participants are concerned about sustaining their overall health; and they are used to living their lives, using their bodies, and managing the limitations of their bodies. Providers need multipurpose tools that provide education about DXA and osteoporosis medication, and that also target patients' multilayered beliefs about DXA and osteoporosis medication. For example, providers need to be able to engage beliefs about how the bone health medications are dangerous or ineffective *and* the belief that fractures are from bad luck *and* the belief that osteoporosis is a disease that primarily affects women. Work to improve care of this population needs to recognize that bone health providers are not adding a behavior of medication taking to patients, they are changing a behavior or belief.

Our study has several limitations. We contacted potential participants 9 to 12 months after their care decisions with the RBHT, which may have heightened recall bias. Some participants also expressed confusion in understanding the difference between the letters they received about participating in the RBHT and the letters they received about participating in our research study. We did not collect demographic information (e.g., race, age, education, comorbidities), though this information would be helpful in making future claims about generalizability. When this study was conducted, there was little information on patient‐level barriers to treatment, and so the interviews focused specifically on participants' decision‐making. Finally, we did not use any survey measures of knowledge or beliefs, so we are not able to categorize our qualitative data according to these scales; if we had used these scales, it would be easier to compare our findings to findings from other studies. Even so, our data provide some information about how patients currently access care (i.e., osteoporosis care pathways) and why patients decline osteoporosis care.

Our findings inform the ongoing implementation of telemedicine clinics developed to serve geographically dispersed, older adult populations, and contribute new information about patient barriers to care. Focusing on medications may limit opportunities for care providers to stay in conversation with their patients and work with them to manage their bone health care.

## AUTHOR CONTRIBUTIONS

**Jen Van Tiem:** Formal analysis; writing‐original draft; writing‐review & editing. **Melissa Steffen:** Data curation; formal analysis; writing‐review & editing. **Aaron Seaman:** Data curation; writing‐review & editing. **Karla Miller:** Writing‐review & editing. **Shylo Wardyn:** Data curation; project administration. **Chris Richards:** Data curation; writing‐review & editing. **Samantha Solimeo:** Conceptualization; formal analysis; funding acquisition; investigation; methodology; supervision; writing‐review & editing.

## DISCLOSURES

JMVT reports no disclosures of potential conflicts of interest. MJAS reports no disclosures of potential conflicts of interest. ATS reports no disclosures of potential conflicts of interest. KM reports no disclosures of potential conflicts of interest. SEW reports no disclosures of potential conflicts of interest. CCR reports no disclosures of potential conflicts of interest. SLS reports no disclosures of potential conflicts of interest.

### PEER REVIEW

The peer review history for this article is available at https://publons.com/publon/10.1002/jbm4.10501.

## Supporting information


**Appendix S1** Interview Guide

## Data Availability

Research data are not shared due to privacy/ethical restrictions.
