# Supplementary material for: Attitudes toward bone health among rural‐dwelling veterans identified as at risk of fracture: a qualitative analysis
Source: JBMR Plus. 2021 May 14;5(6):e10501. doi: 10.1002/jbm4.10501 (PMC8216131; doi:10.1002/jbm4.10501)
Supplement: Supplementary file 1 — Appendix S1 Interview Guide [file JBM4-5-e10501-s001.docx]

Patient Interview Questions

| - - - 1. When you got the letter or phone call from the bone health team telling you that you might be at risk of bone problems­--what did you make of that news? *What did you do?*   *If treated group go to item 2, if not treated go to items 7, 10, 11*   1. Why did you decide to have a DXA, the bone scan? 2. How did you get the bone scan results? 3. What was your reaction? 4. What did the bone health team suggest that you do to take care of your bones? 5. Did you decide to [take medication, go to physical therapy, etc.?] 6. Could you explain what osteoporosis is, in your own words? 7. Do you have any ideas as to why you happened to develop osteoporosis? 8. How long do you think you’ll have this? 9. Is osteoporosis a health problem you think is important? 10. Are you generally happy with the care that you receive at the VA? |
| --- |
